# Designing and fabrication of curcumin loaded PCL/PVA multi-layer nanofibrous electrospun structures as active wound dressing

**DOI:** 10.1007/s40204-017-0062-1

**Published:** 2017-02-02

**Authors:** Seyed Mahdi Saeed, Hamid Mirzadeh, Mojgan Zandi, Jalal Barzin

**Affiliations:** 1 Department of Biomaterials, Iran Polymer and Petrochemical Institute, Tehran, Iran; 2 Department of Polymer Engineering and Color Technology, Amirkabir University of Technology, Tehran, Iran

**Keywords:** Electrospinning, Nanofibers, Wound dressing, Response surface

## Abstract

Active wound dressings play a significant role in burn and chronic wound treatment. In this study, electrospinning process is used to fabricate a novel three-layer active wound dressing based on ε-polycaprolactone (PCL), polyvinylalcohol (PVA), and curcumin (CU) as a biologically active compound. The main purpose for developing such a system is to control wound exudates, which remains a challenge, as well as enjoying the anti-bacterial property. Electrospinning process parameters are optimized by response surface methodology to achieve appropriate nanofibrous electrospun mats, and then, a three-layer dressing has been designed in view of water absorbability, anti-bacterial, and biocompatibility characteristics of the final dressing. The results illustrate that a three-layer dressing based on PCL/curcumin containing PVA as a middle layer with optimized thickness which is placed over the incision, absorbs three times exudates in comparison with pristine dressing. Anti-bacterial tests reveal that the dressing containing 16% curcumin exhibits anti-bacterial activity without sacrificing the acceptable level of cell viability.

## Introduction

Burn injury is highly common worldwide with a high annual casualty rate. The most severe kind of burn injury is that of third-degree burn with a high risk of death; and there are two major problems with third-degree burn patients. First, a vast area of skin is damaged; consequently, the body is easily attacked by microorganisms, most specifically bacteria (Lee et al. 2006). Second, wound exudates cover the wound as a result of normal behaviour of immunity system. The accumulation of the exudates around the wound may increase the risk of wound infection and adjacent organs and also delay the wound healing process (Church et al. 2006; Pruitt et al. 1998). Hence, these two essential problems need to be taken into consideration to design active wound dressings.

Moreover, a good dressing should provide minimum adherence to the wound surface to be easily released from the wound bed after recovery without causing further tissue injuries (Pankhurst and Pochkhanawala 2002). Therefore, an active wound dressing should have proper mechanical properties, desirable flexibility, optimal water uptake, easy application, and reasonable price, as well as anti-bacterial activity.

Curcumin is a yellow dyestuff and active constituent of turmeric. For centuries, turmeric has been used to treat several diseases in the traditional and herbal medicine of south and Southeast Asia. For the same reason, medicinal properties of curcumin have attracted the attention of many scientists during the last three decades (Aggarwal et al. 2007). So far, many unique properties of curcumin are recognized to be used for medical purposes; including: anti-oxidant, anti-inflammation, and anti-infective properties (Chainani-Wu 2003; Prasad et al. 2014). These properties have established curcumin as a unique material for wound healing and treatment of inflammatory diseases.

Despite all biologic properties, curcumin is extremely unstable in vivo with a very low bioavailability (Anand et al. 2007). As it has been revealed in the previous studies, uptake of 450–3600 mg edible curcumin demonstrates a very low bioavailability in patient (Hsu and Cheng 2007). For the same reason, many studies have been carried out to improve the bioavailability of curcumin using different drug carriers to obviate this problem (Anand et al. 2007, 2010; Foltran et al. 2008; Sasaki et al. 2011; Shaikh et al. 2009).

ε-Polycaprolactone is a biodegradable polymer vastly studied for various fields of tissue regeneration and wound healing (Merrell et al. 2009; Saeed et al. 2015). Medical applications of nanofibrous matrices have also been greatly addressed by scientists because of their abilities in imitating the physical form of collagen fibrils existing in natural ECM in the body (Ramakrishna 2005). High surface area of nanofibrous matrices increases the interaction between the wound dressing and tissue and it facilitates the release of loaded bioactive molecules (Ramakrishna et al. 2006). Different solution, process, and environmental parameters may affect the spinning ability and fiber morphology of the electrospun mats. Since many process parameters may influence the electrospinning method, different methods, such as group method of data handling (GMDH), artificial neural network (ANN), and response surface methodology (RSM), were used to improve, regulate, and optimize the electrospinning conditions (Bhardwaj and Kundu 2010; Chaudhuri et al. 2013; Chomachayi et al. 2016; Tsimpliaraki et al. 2011).

Gu et al. evaluated the quantitative relationship between electrospinning process parameters and the average size and distribution of fibers by RSM (Gu et al. 2005). Pezeshki et al. assessed the effect of blend ratio, applied voltage, and flow rate on the standard deviation of fiber diameter (SDF) and mean fiber diameter (MFD) of gelatin/chitosan mats (Pezeshki‐Modaress et al. 2015a, b).In another study, these researchers optimized the gelatine/chondroitin sulfate (CS) electrospun mats by RSM (Pezeshki-Modaress et al. 2015a, b).

This study constitutes the design and fabrication of an active dressing to be used in third-degree burn injuries. Considering the required properties for such dressing, polycaprolactone is used as the main component of dressing, because, apart from its biocompatibility, it also enjoys desirable mechanical properties and flexibility. Moreover, it is hydrophobic with minimum adherence to the wound. On the other hand, the benefits of a substance, such as curcumin, are because of its anti-bacterial and anti-inflammatory properties. To manage the exudates, a median layer of polyvinyl alcohol is used to absorb the exudates. Finally, a three-layer dressing having curcumin was designed and fabricated. The results show that non-woven electrospun wound dressing based on PCL/PVA/PCL achieve the excellent benefits, such as soft, conformable, non-adhesive, easy to cover the wounds with irregular shapes, as well as anti-bacterial characteristics.

## Experimental

### Materials

PCL with molar mass of 80 kDa, which was used as the main component of the dressing guaranteeing desirable physical and mechanical properties, was supplied from Sigma-Aldrich, Germany. PVA with molar mass of 85–124 kDa as a moisture (exudates) absorber and curcumin (CU) as an active anti-bacterial and anti-inflammatory material were supplied from Sigma-Aldrich, Germany. All solvents, including dimethylformamide (DMF), dichloromethane (DCM), chloroform, ethanol, and methanol, were supplied by Merck Co., Germany. Fibroblastic cell line (L929), *Escherichia coli,* and *Staphylococcus aureus* were obtained from National Cell Bank, Pasteur Institute of Iran.

### Solution preparation

To provide a three-layer wound dressing, the first step involved the preparation of PVA and PCL solutions. To prepare 10 ml PVA solution, 800 mg of this material was added into 10 ml of 90 °C water and stirred for 3 h, and then the solution was cooled to ambient temperature.

To prepare PCL and PCL/CU solutions, a mixed solvent system of DMF/DCM was prepared by 2/1 volumetric ratio and 1.25 g of PCL was added into 10 ml of the mixed solvent and stirred for 4 h to prepare 12.5% PCL solution. In the next step, a PCL/CU solution was prepared by certain amount of curcumin added into the 12.5% PCL solution and stirred for 2 h to prepare PCL/CU solutions.

### Electrospinning process

Electrospinning machine model CO881007NYI manufactured by Asian Nanostructures Technology Company, Iran was used to prepare nanofiber mats. This machine was equipped with drum collector (50 × 70 mm).

The main purpose of this study was design and fabrication of a three-layer nanofiber wound dressing using electrospun-based PCL and PVA. Here, PCL was used as the main phase of wound dressing to guarantee desirable mechanical properties as well as the feature of non-adherence to the wound. A layer of PVA was also used in the median layer to absorb exudates.

**Fig. 1:**
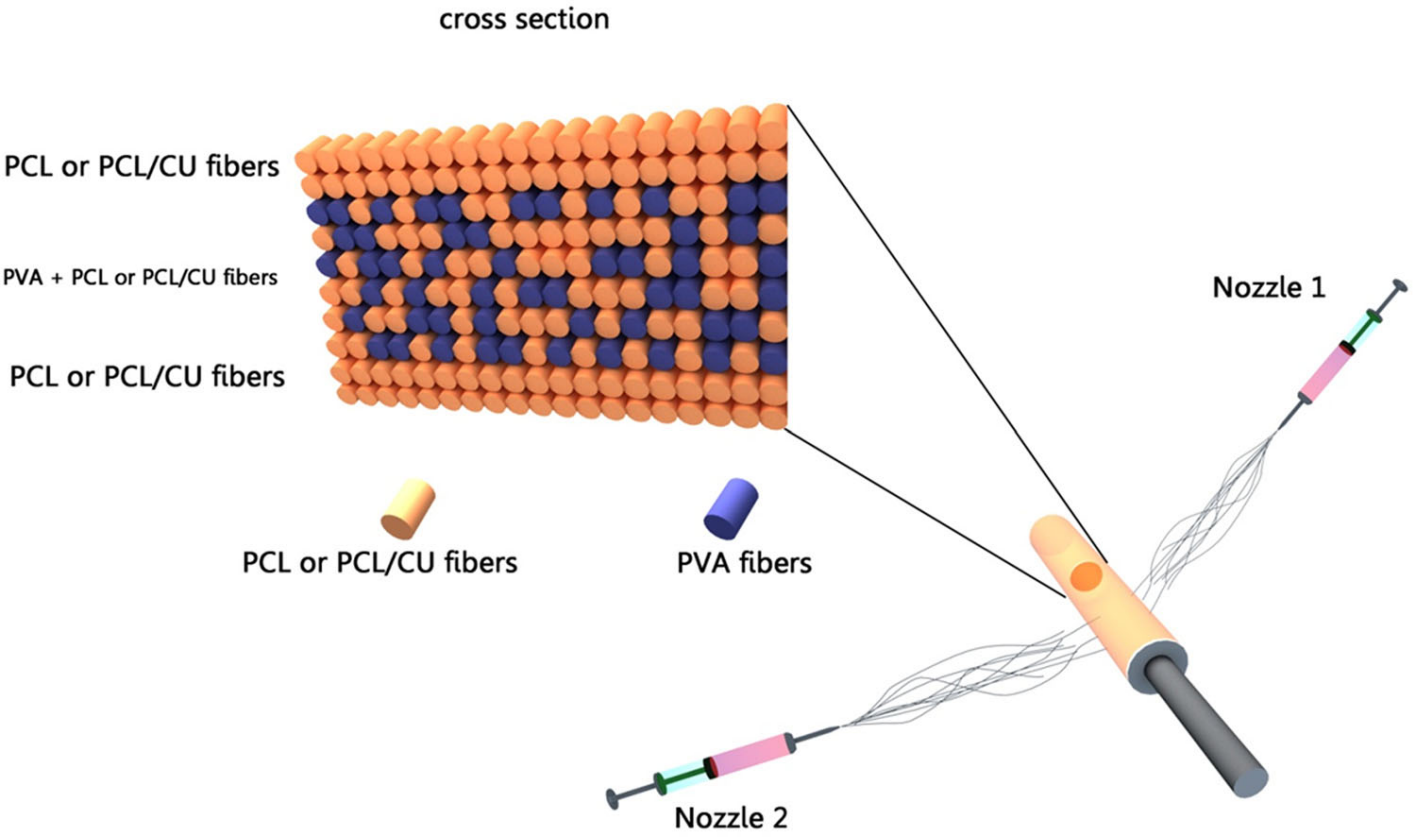
Profile of co-electrospinning process

As stated earlier, curcumin was used in this wound dressing to ensure anti-bacterial and inflammatory properties which every active dressing should possess. Since the anti-bacterial factor should be in direct contact with the wound to perform its role, the nanofibrous outer layer of each PCL specimen was loaded with curcumin of different percentage. Therefore, nanofiber mats of PCL, PVA, and PCL loaded with curcumin were optimized on the basis of response surface methodology (RSM) modeling. The three-layer dressings were prepared after optimization of the nanofiber mats by RSM and obtaining solution conditions to achieve suitable process for mats formation with desirable morphology. For this purpose, a two-nozzle electrospinning method was applied (Fig. 1). As it can be observed in Fig. 1, PCL solution (or PCL loaded with curcumin) is injected using nozzle 1 and it is electrospun for 15 min; then, nozzle 2 (PVA) is started to inject simultaneously. After 3 h of PVA injection, nozzle 2 stops injecting and nozzle 1 keeps spinning PVA fibers for 15 min. In the end, the product constituting a three-layer dressing was cut into desirable sizes for several characterization tests.

### Response surface

To optimize the morphology of different mats in this study, quadratic model of Box-Behnken design in response surface methodology was employed to establish experiential relationships between three electrospinning parameters (curcumin to PCL ratio, applied voltage, and the flow rate) at three equally spaced levels of each factor and for number of beads per unit area response using the Design-Expert 7.0 software. According to the best of our knowledge, there is no previous report on morphology optimization of number of beads per unit area in nanofibrous active dressings by RSM which is important in specific applications. There are several reports on fiber diameter and standard deviation of fiber diameter optimization by RSM (Pezeshki‐Modaress et al. 2015a, b). In this study, we selected a solution system that the above two responses still remained almost constant. In all the experiments, the working distance, rotating velocity of the drum, angle of the nozzle in relation to the collector, and PCL concentration were set at 160 mm, 250 RPM, 90^o^, and 12.5%, respectively. The following parameters were varied at three levels during electrospinning: CU/PCL ratio 1, 8.5, 16%; applied voltage 12, 18, 24 kV; flow rate 1, 2, 3 mL h^−1^. The morphology and bead number of electrospun nanofibers were investigated using SEM (VEGA, TESCAN, Czech Republic) after gold sputter coating. The bead number of the electrospun nanofibers was measured by counting the number of beads located on a square millimeter. Table 1 shows the parametric levels of the experimental design and related bead numbers.

**Table 1:** Experimental design and response of nanofibers for three factors at three levels

Experiment number	*C* CU ratio (%)	*V* applied voltage (kV)	*F* flow rate (mL/hr)	Response: beads number per unit area (mm^−2^)
1	1	12	2	3573
2	16	12	2	5763
3	1	24	2	3799
4	16	24	2	5433
5	1	18	1	965
6	16	18	1	1998
7	1	18	3	4111
8	16	18	3	7299
9	8.5	12	1	2376
10	8.5	24	1	2589
11	8.5	12	3	8749
12	8.5	24	3	9471
13	8.5	18	2	3678
14	8.5	18	2	3050
15	8.5	18	2	3056
16	8.5	18	2	3029
17	8.5	18	2	3077

### Microscopy

Scanning electron microscopy (SEM) model VEGA II manufactured by TESCAN from Czech was used to analyze the morphology and fine-structure of the electrospun nanofibrous wound dressings.

Optical microscope (T-B2.5 X, Japan, Nikon) was used to observe the growth process of the fibroblasts cultured on wound dressings.

### Water absorption, transmission, and contact angle

Water absorption tests were carried out to measure the capacity of the optimized fabricated wound dressings in absorbing exudates. Hence, 2 × 2 cm pieces of different wound dressings were weighed and soaked in water. The samples were weighed at 5 min intervals (30 min) to acquire a constant weight for each sample. Then, the samples were reweighed to obtain the water absorption rate for the dressings (Modaress et al. 2012). Here, the electrospun PCL mat specimen was used as test control.

To measure the water vapour transmission rate (WVTR) of the samples, mats were fixed at the circular opening of a transmission bottle (0.8 cm diameter and 5 cm height), which were stored in germinators under relative humidity of 40% at 25 °C. The weight of each bottle with water was measured at different time intervals and the WVTR of the mat was obtained by the following definition:

1 $$ {\text{WVTR}} = \frac{ - \Delta W}{A \times \Delta t} $$

where Δ*W* as the change in water weight, *A* designating the exposure area of the film, and Δ*t* as the exposure time (Zahedi et al. 2010).

The dynamic water contact angle was used to further explore absorption of water by the mats. This evaluation was carried out by G10 device made by KRUSS Germany.

### MTT assay

MTT assay was used to study the viability of L929 cells exposed to several prepared electrospun dressings. For this purpose, L929 cells were cultured according to ISO 10993-5 on different wound dressings. The L929 cells were placed in growth media (RPMI-1640) supplemented with 10% FBS. The cells were put in an incubator and incubated at 37 °C, 5% CO_2_, and 90% humidity. The scaffold was sterilized by UV (the sterilization time was 20 min at 15 cm) before cell seeding. The samples were kept in the incubator for 48 h. After 48 h, the cells were counted by ELISA reader ELX 808. Cytotoxicity was rated based on cell viability (after 48 h) relative to controls as: non-cytotoxicity >90% cell viability, slightly cytotoxicity¼ 60–90% cell viability, moderately cytotoxicity¼ 30–59% cell viability, and severely cytotoxic ¼ <30% cell viability (Badole et al. 2013; Dahl et al. 2006).

### Anti-bacterial test

Anti-bacterial test measures the efficiency of manufactured dressings in killing Gram-negative and Gram-positive bacteria. For this reason, the anti-bacterial activities of wound dresses were investigated against model microbial species, including *Escherichia coli* (Gram-negative) and *Staphylococcus aureus* (Gram-positive), through the following method.

In this method, wound dresses were cut into 1 × 1 cm^2^ and sterilized by UV radiation for 15 min. Next, 5 mL of growth medium (1500 cfu containing Luria–Bertani medium) for each bacterial species was taken in sample-contained tube. The tubes were then seeded with 1 mL fresh culture of bacterial strains and incubated in a shaking incubator at 37 °C and for 48 h. The turbidity of the media was observed after 48 h at 610 nm using a UV spectrophotometer (spectrophotometer Epoch, Biotek instruments, U.S.) and the numbers of survived bacteria were calculated by calibration curve.

## Results and discussion

As its main objective, this study was focused on the design and fabrication of active wound dressing. An active wound dressing was based on PCL and PVA containing curcumin which was designed and fabricated using electrospinning technique. The active three-layer wound dressings were fabricated which were capable to manage exudates, provide an optimal wound moist, and control the wound exudates which is necessary for wound healing, as well as killing or keeping away the surrounding microorganisms. PVA acts as a moisture absorber and the anti-bacterial efficacy of curcumin was used to kill Gram-positive and Gram-negative bacteria.

### Optimization of PCL and PVA mats

To optimize the fiber morphology of PCL, the number of beads was minimized in the mats to achieve a desirable morphology by changing various procedural and solution-related variables. As shown in Table 1, in this study, the effect of curcumin ratio, applied voltage, and flow rate on beads number was investigated. RSM was used to develop deeper understanding of the process parameters and inaugurate a quantitative equation between the electrospinning process parameters and beads number (Gu and Ren 2005). *P* values were used for measuring the statistical significance of the model and its parameters. A factor has a significant impact on the response when *P* is less than 0.05. For *P* greater than 0.05, the factor has no significant impact on the response. Another important factor which is used to evaluate the model is *R* ^2^ (Pezeshki‐Modaress et al. 2015a, b). This factor represents the proportion of the total variability that has been explained by the regression model (Gu and Ren 2005).

*R* ^2^ is a criterion for the amount of response variation which is explained by variables and always increases when a new term is added to the model, regardless of whether introducing more terms is statistically significant. Adj-*R* ^2^ is the adjusted form of *R* ^2^ for the number of terms in the model; therefore, it increases only if the new terms improve the model and it decreases if unnecessary terms are added. Pred-*R* ^2^ implies how well the model predicts the response for new observations, whereas *R* ^2^ and Adj-*R* ^2^ indicate how properly the model matches the empirical results (Ziabari et al. 2010).

**Fig. 2:**
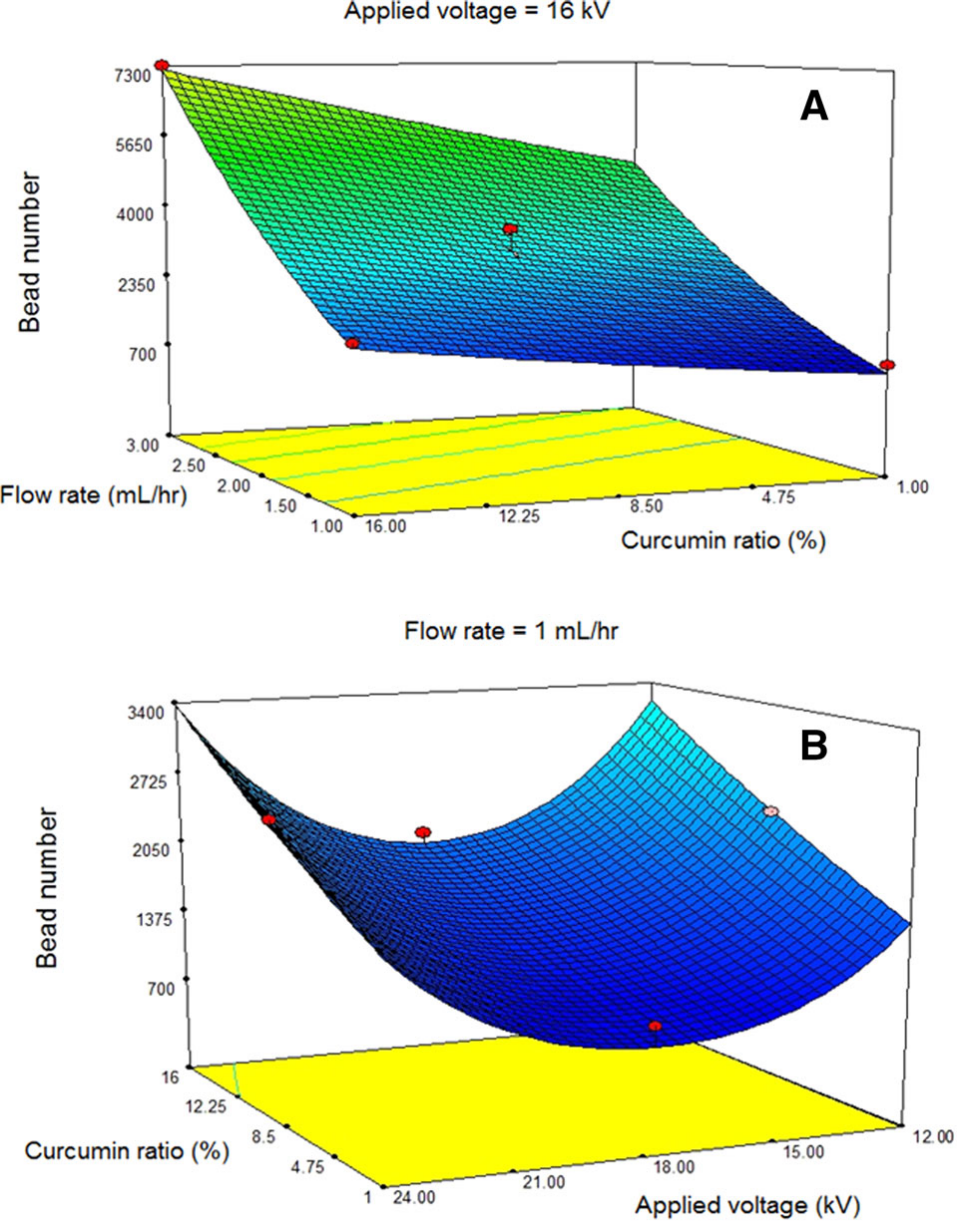
Response surfaces for the beads number (mm^−2^) in terms of **a** flow rate and curcumin ratio and **b** curcumin ratio and applied voltage

Quadratic models with different forms could be used to model the bead numbers. We have used five common equation forms for response surface modeling of the experimental data, and the *P* values, *R* ^2^, Adj-*R* ^2^, and Pred-*R* ^2^ for each equation form were compared. Considering the lowest *P* values and the highest *R* ^2^, Adj-*R* ^2^, and Pred-*R* ^2^ for the square root form, the response surface equation for bead number is given by the following:

2 $$ {\text{Number of beads }} = 128.76571 + \left( {1.05274 \times C} \right){-}\left( {13.74257 \times V} \right) + \left( {20.62707 \times F} \right) + \left( {0.38497 \times V^{2} } \right) $$

where *C* is the curcumin ratio (%) and *V* and *F* are the values of applied voltage (kV) and flow rate (mL/h). As it is mentioned in Table 2, *R* ^2^, Adj-*R* ^2^, and Pred-*R* ^2^ are very suitable for this equation. According to the equation, bead numbers with curcumin ratio and flow rate have a linear relationship, but quadratic function is dominant between the bead number and applied voltage. Figure 2 illustrates 3D graphs to show linear and quadratic relations between the bead number and parameters.

**Table 2:** *R* ^2^, Adj-*R* ^2^ and Pred-*R* ^2^ in square root equation

*R* ^2^	Adj *R* ^2^	Pred *R* ^2^
0.9714	0.9619	0.9339

**Fig. 3:**
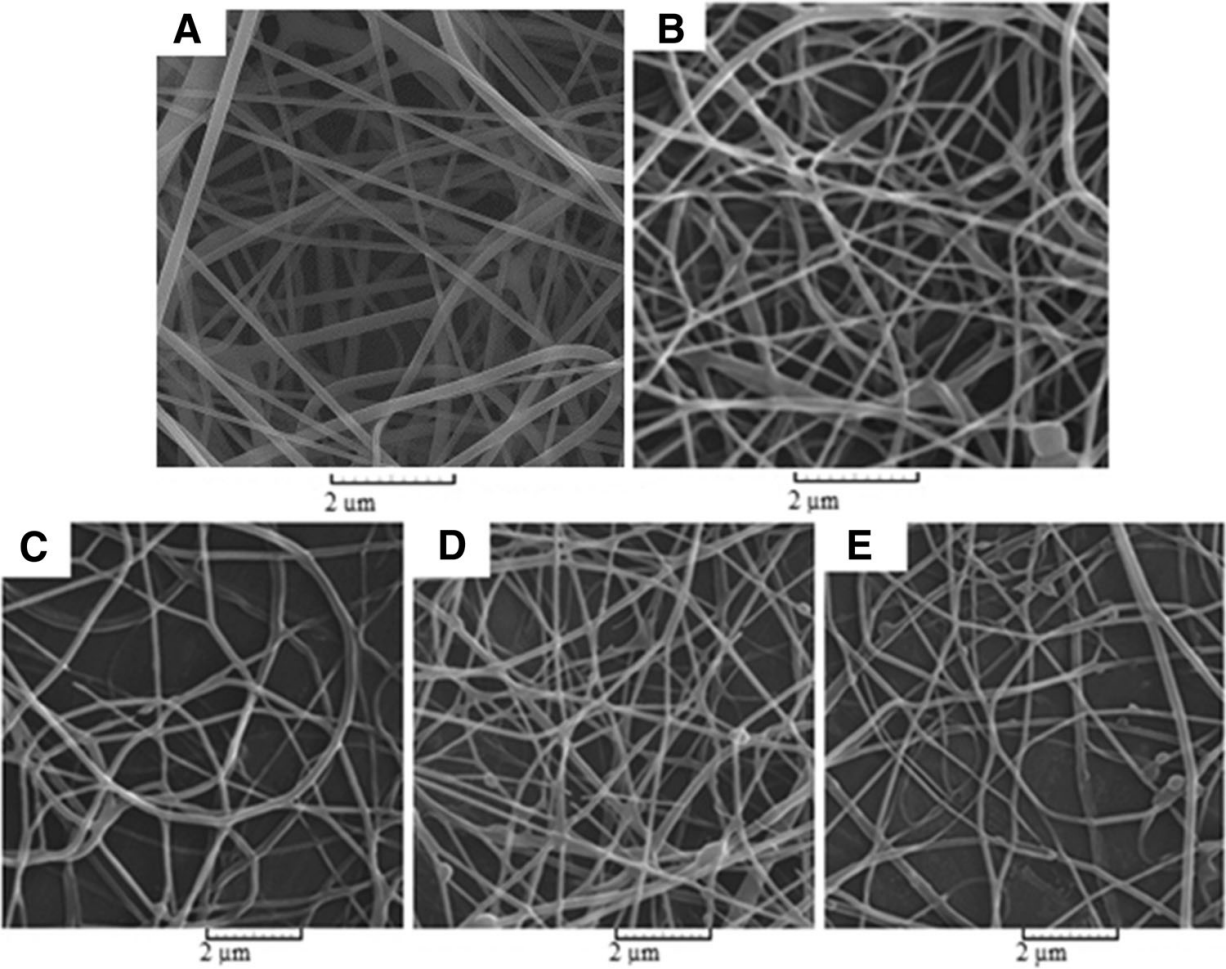
Electron microscope images of optimized mats, **a** PVA, **b** PCL, and PCL containing different curcumin loadings **c** 1%; **d** 8.5%; **e** 16%

According to the equation and by design-expert software, minimization of the bead number was carried out. The optimal conditions, necessary to obtain different mats of the same thickness in the same flow rates in co-electrospinning process, are presented in Table 3. SEM images of different electrospun mats under optimal conditions are also illustrated in Fig. 3.

**Table 3:** Optimized electrospinning parameters for mats with different curcumin loadings

Mat composition	Parameters			
	Feeding rate (mL h^−1^)	Working distance (mm)	∆*V* (kV)	Polymer. conc. (wt%)
PCL	1	160	18.4	12.5
PCL/1% CU	1	160	18	12.5
PCL/8.5% CU	1	160	17.8	12.5
PCL/16% CU	1	160	17.8	12.5

Table 4 represents a three-layer wound dressing with different curcumin loadings fabricated and compared with a system without curcumin and a system coated with curcumin only. Water absorption, biocompatibility, and anti-bacterial properties of these different categories are evaluated and discussed.

**Table 4:** Different classifications of three-layer wound dressings for different tests

Sample	Layer composition	CU/PCL ratio (%)
0%	PCL/PVA/PCL	0
Coated	PCL/PVA/PCL	Coated with curcumin
1%	PCL/PVA/PCL	1
8.5%	PCL/PVA/PCL	8.5
16%	PCL/PVA/PCL	16
Reference 1	PCL	0
Reference 2	Tissue culture plate	0

### Water absorbability and transmission rate

**Fig. 4:**
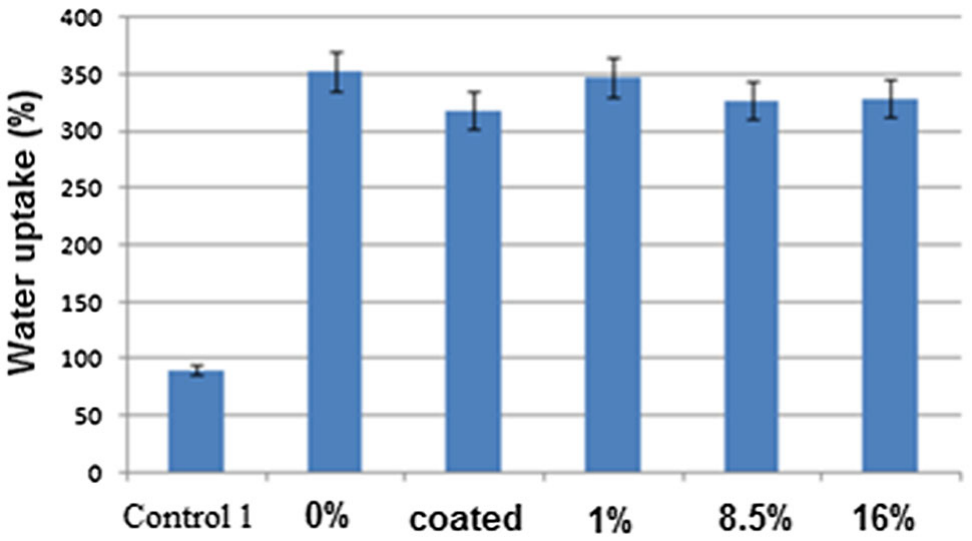
Water absorption rate of different wound dressings

Maintaining an appropriate level of humidity between a wound dressing and a wound is critical, because a failure to control exudates can result in dry wound or wound infection. As a result, the ability to control the moisture balance between the wound and wound dressing is a key aspect of wound healing. Figure 4 shows the absorption rate of mats with different compositions.

As it is illustrated in Fig. 4, water absorption rate is increased up to three times, when a PVA containing layer is mounted as a middle layer in the wound dressings. This indicates the efficiency of three-layer wound dressings to exudates absorption; on the other hand, it can be observed that curcumin percentage and loading method cause no significant change in water absorption rate. Hence, it can be suggested that three-layer wound dressings enjoy a higher potential in exudates absorption by leading them to the outer layer of dressing, as compared to one-layer type dressing. For the same reason, accumulation of exudates beneath the wound dressings decreases and this can help the recovery process of a wound within a shorter period of time.

**Fig. 5:**
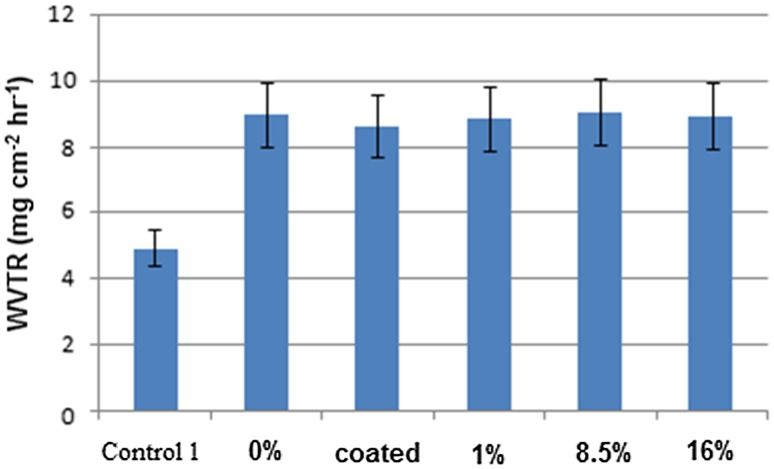
Water vapour transmission rate of wound dressings with different CU%

Usually, for normal skin, WVTR is ~0.85 mg cm^−2^ h^−1^, and for an injured skin, it is 1.16–21.41 mg cm^−2^ h^−1^. A wound dressing material must have a suitable WVTR to prevent additional dehydration and exudation. Therefore, a wound bandage with WVTR in the range of 8.33–10.42 mg cm^−2^ h^−1^ is suggested (Zahedi et al. 2010). Figure 5 shows WVTR values of different kinds of mats. As compared to the control sample (monolayer mat), all the films show increased WVTR values because of the high hydrophilicity potential of PVA layers and reduced resistance against the permeability of water molecules.

### Contact angle

**Fig. 6:**
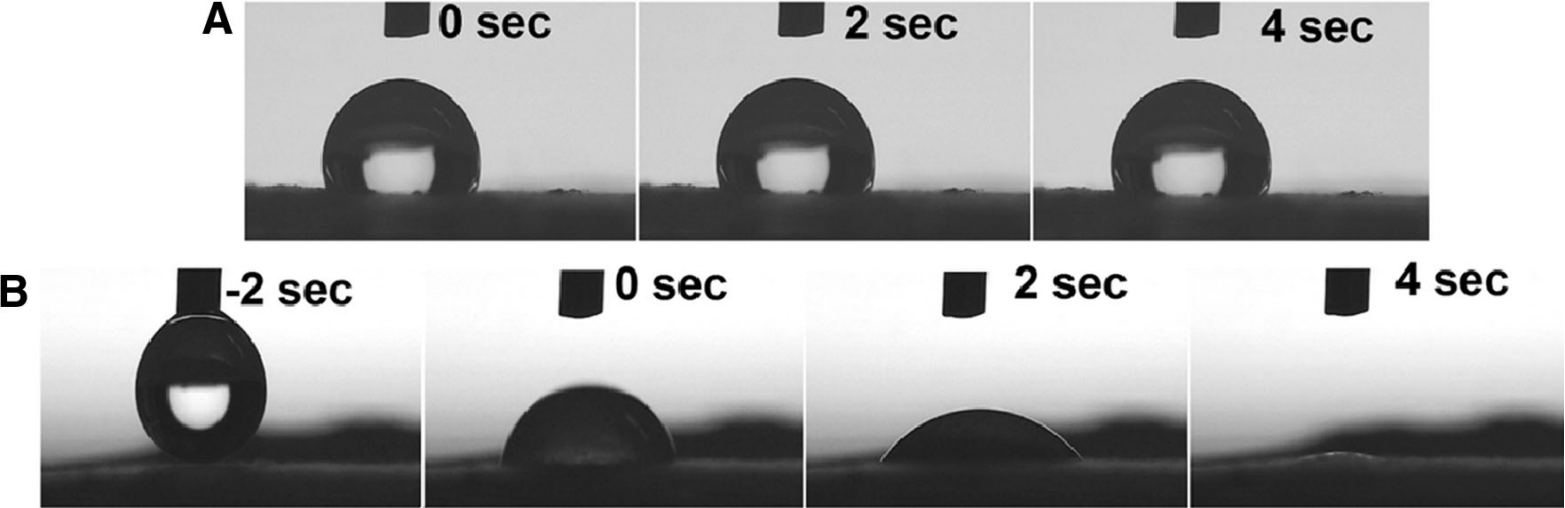
Dynamic contact angle: **a** monolayer PCL and **b** three-layer PCL/PVA/PCL

The water contact angle is another criterion to assess wound dressings. Figure 6 shows contact angle values of monolayer PCL and three-layer PCL/PVA/PCL mats during the time period; as it is already mentioned, a three-layer wound dressing displays a dynamic behaviour and could absorb water droplet quite unlike the monolayer mat which reveals super hydrophobic behaviour. This confirmed the high performance of PVA layer in exudates absorption rate.

### Anti-bacterial properties

**Fig. 7:**
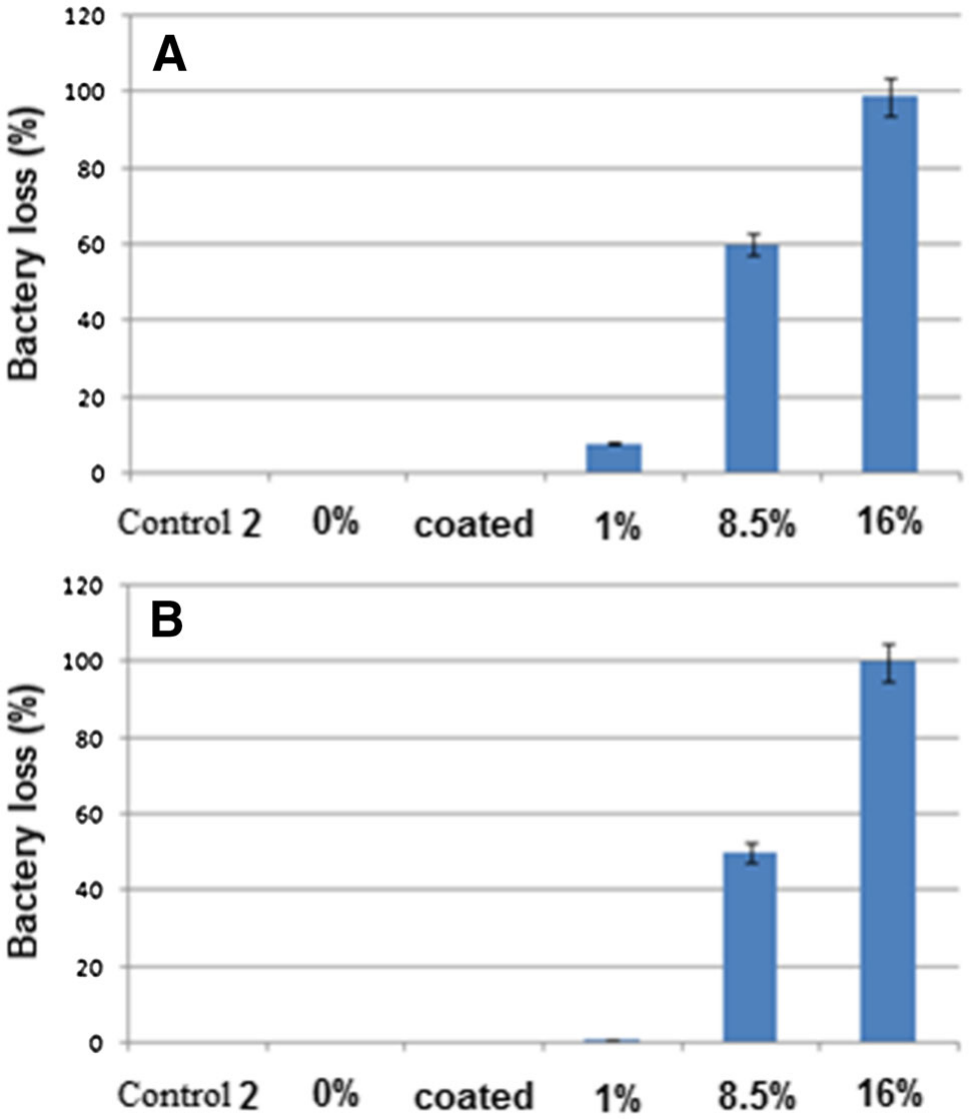
Anti-bacterial properties for different CU% wound dressings: **a** *Staphylococcus Aureus* and **b** *Escherichia coli*

Anti-bacterial testing is used to measure the efficiency of three-layer dressings in curcumin release and to evaluate their activity to kill microorganisms. For this purpose, two bacteria of *E. coli* (as Gram-negative bacteria) and *S. Aureus* (as Gram-positive bacteria) were cultured on the obtained electrospun wound dressings. The survived bacteria remaining on the culture were counted after 48 h. The percentage of killed bacteria indicated the efficiency of three-layer wound dressings in killing the bacteria, i.e., the anti-bacterial properties of the dressings. Figure 7 shows the percentage of bacteria reduction after 48 h exposure against various dressings.

Referring to the diagram, it can be observed that the dressings show the same bacterial activity against both Gram-positive and Gram-negative bacteria. Moreover, the dressings with less than 8.5% curcumin do not reveal significant anti-bacterial properties. Though the three-layer wound dressings, loaded with more than 8.5% curcumin, illustrate desirable anti-bacterial properties, dressings containing 16% curcumin are capable of killing 100% of bacteria after 48 h. Dressings (4) and (5) can be suggested as appropriate for burn recovery, if they are not significantly toxic. For the same reason, L929 fibroblast cell line was used to assess the toxicity of three-layer dressings fabricated by electrospinning technique.

### Biocompatibility

**Fig. 8:**
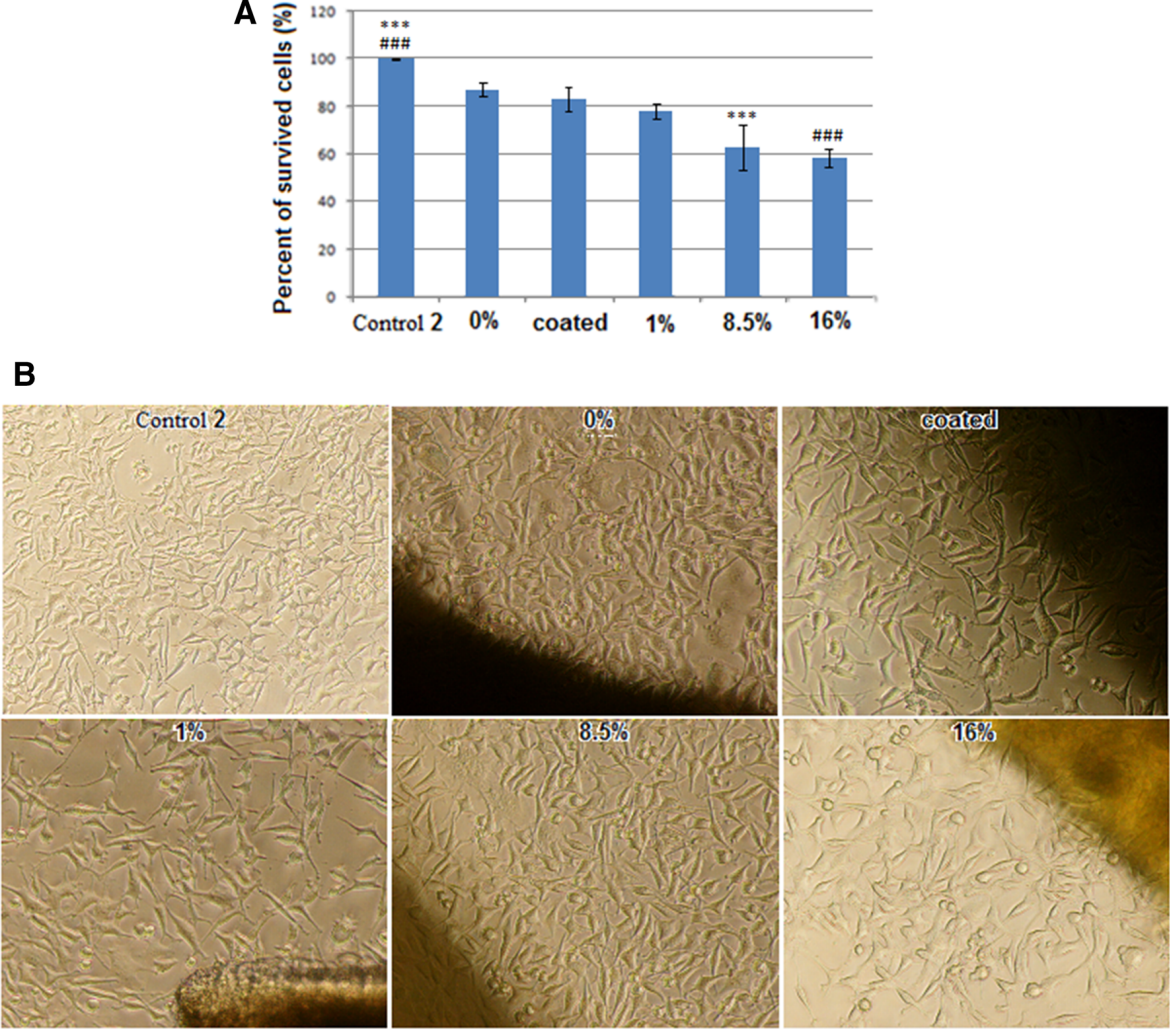
MTT assay results (**a**) and cell images (**b**) for L929 cell line on different CU % dressings after 48 h. Statistical significance was determined by one-way ANOVA, and values of *P* < 0.05 were considered statistically significant

Safety evaluation study (in vitro) was conducted on a variety of fabricated active wound dressings to identify the presence of toxicity or any potential harmful species. To study the cell behaviour, L929 cell lines were cultured on fabricated wound dressings and the percentage of cells surviving after 48 h was reported. The results of this test are illustrated in Fig. 8.

As it can be seen, curcumin has the ability to exert anti-bacterial and it increases in toxication of the dressings. However, active wound dressing containing 16% curcumin with the highest toxicity reveals ~60% of cell survival after 48 h. These observations show that the dressing containing curcumin, despite anti-bacterial properties, maintains slight toxicity with its biocompatibility. Therefore, we may conclude that 16% curcumin active dressing keeps a proper balance between toxicity and anti-bactericity.

## Conclusion

Controlling the moisture balance between the wound and wound dressing is a key aspect of wound healing. In this research, an active wound dressing with appropriate level of humidity was designed. Electron microscope images showed that different classifications of dressings are well optimized with regard to the presence of bead by RSM. Moreover, the results of absorbability test revealed that the addition of a PVA layer increases the absorbability by three times; this indicates the efficiency of this dressing layer toward absorption of exudates.

The results of anti-bacterial test indicated that minimum load of curcumin with the potential to kill Gram-positive and Gram-negative bacteria is 8.5% of the weight proportionate to the PCL. As curcumin load of the dressings increased, the anti-bacterial properties also increased. The dressing with 16% load of curcumin killed all Gram-positive and Gram-negative bacteria after 48 h, and this sample was considered as an appropriate one concerning the anti-bacterial properties.

The results of biocompatibility test also showed that as curcumin load increased, the viability of fibroblast cells decreased; however, the viability rate of cells on the sample loaded with 16% curcumin was a satisfactory rate of 58%. According to the results, it can be suggested that, concerning anti-bacterial properties as well as the viability of fibroblast cells, dressings loaded with 16% curcumin are an ideal formulation. The sample loaded with 8.5% curcumin is the second candidate.

## References

[CR1] Aggarwal BB, Sundaram C, Malani N, Ichikawa H (2007). Curcumin: the Indian solid gold, the molecular targets and therapeutic uses of curcumin in health and disease.

[CR2] Anand P, Kunnumakkara AB, Newman RA, Aggarwal BB (2007). Bioavailability of curcumin: problems and promises. Mol Pharm.

[CR3] Anand P, Nair HB, Sung B, Kunnumakkara AB, Yadav VR, Tekmal RR, Aggarwal BB (2010). Design of curcumin-loaded PLGA nanoparticles formulation with enhanced cellular uptake, and increased bioactivity in vitro and superior bioavailability in vivo. Biochem Pharm.

[CR4] Badole GP, Warhadpande MM, Meshram GK, Bahadure RN, Tawani SG, Tawani G, Badole SG (2013). A comparative evaluation of cytotoxicity of root canal sealers: an in vitro study. Restor Dent Endod.

[CR5] Bhardwaj N, Kundu SC (2010). Electrospinning: a fascinating fiber fabrication technique. Biotech Adv.

[CR6] Chainani-Wu N (2003). Safety and anti-inflammatory activity of curcumin: a component of tumeric (*Curcuma longa*). J Altern Comp Med.

[CR7] Chaudhuri S, Chakraborty R, Bhattacharya P (2013). Optimization of biodegradation of natural fiber (*Chorchorus capsularis*): HDPE composite using response surface methodology. Iran Polym J.

[CR8] Chomachayi MD, Solouk A, Mirzadeh H (2016). Electrospun silk-based nanofibrous scaffolds: fiber diameter and oxygen transfer. Prog Biomater.

[CR9] Church D, Elsayed S, Reid O, Winston B, Lindsay R (2006). Burn wound infections. Clin Microbiol Rev.

[CR10] Dahl JE, Frangou-Polyzois MJ, Polyzois GL (2006). In vitro biocompatibility of denture relining materials. Gerodontology.

[CR11] Foltran I, Foresti E, Parma B., Sabatino P, Roveri N (2008) Novel biologically inspired collagen nanofibers reconstituted by electrospinning method. Paper presented at the Macromol Symp

[CR12] Gu SY, Ren J (2005). Process optimization and empirical modeling for electrospunpoly (D, L-lactide) fibers using response surface methodology. Macromol Mater Eng.

[CR13] Gu S, Ren J, Vancso G (2005). Process optimization and empirical modeling for electrospun polyacrylonitrile (PAN) nanofiber precursor of carbon nanofibers. Eur Polym J.

[CR14] Hsu CH, Cheng A-L (2007). Clinical studies with curcumin, the molecular targets and therapeutic uses of curcumin in health and disease.

[CR15] Lee SH, Jeong SK, Ahn SK (2006). An update of the defensive barrier function of skin. Yonsei Med J.

[CR16] Merrell JG, McLaughlin SW, Tie L, Laurencin CT, Chen AF, Nair LS (2009). Curcumin-loaded poly (ε-caprolactone) nanofibres: diabetic wound dressing with anti-oxidant and anti-inflammatory properties. Clin Exp Pharm Physiol.

[CR17] Modaress MP, Mirzadeh H, Zandi M (2012). Fabrication of a porous wall and higher interconnectivity scaffold comprising gelatin/chitosan via combination of salt-leaching and lyophilization methods. Iran Polym J.

[CR18] Pankhurst S, Pochkhanawala T, Bousfield CB (2002). Wound care. Burn trauma. Management & nursing care.

[CR19] Pezeshki-Modaress M, Mirzadeh H, Zandi M (2015). Gelatin–GAG electrospun nanofibrous scaffold for skin tissue engineering: fabrication and modeling of process parameters. Mater Sci Eng C.

[CR20] Pezeshki-Modaress M, Zandi M, Mirzadeh H (2015). Fabrication of gelatin/chitosan nanofibrous scaffold: process optimization and empirical modeling. Polym Int.

[CR21] Prasad S, Tyagi AK, Aggarwal BB (2014). Recent developments in delivery, bioavailability, absorption and metabolism of curcumin: the golden pigment from golden spice. Cancer Res Treat Off J Korean Cancer Assoc.

[CR22] Pruitt BA, McManus AT, Kim SH, Goodwin CW (1998). Burn wound infections: current status. World J Surg.

[CR23] Ramakrishna S (2005). An introduction to electrospinning and nanofibers.

[CR24] Ramakrishna S, Fujihara K, Teo W-E, Yong T, Ma Z, Ramaseshan R (2006). Electrospun nanofibers: solving global issues. Mater Today.

[CR25] Saeed M, Mirzadeh H, Zandi M, Irani S, Barzin J (2015). Rationalization of specific structure formation in electrospinning process: study on nano-fibrous PCL-and PLGA-based scaffolds. J Biomed Mat Res Part A.

[CR26] Sasaki H, Sunagawa Y, Takahashi K, Imaizumi A, Fukuda H, Hashimoto T, Fujita M (2011). Innovative preparation of curcumin for improved oral bioavailability. Biol Pharm Bull.

[CR27] Shaikh J, Ankola D, Beniwal V, Singh D, Kumar MR (2009). Nanoparticle encapsulation improves oral bioavailability of curcumin by at least 9-fold when compared to curcumin administered with piperine as absorption enhancer. Eur J Pharm Sci.

[CR28] Tsimpliaraki A, Zuburtikudis I, Marras SI, Panayiotou C (2011). Optimizing the nanofibrous morphology of electrospun poly [(butylene succinate)-co-(butylene adipate)]/clay nanocomposites and revealing the effect of the fibre nano-dimension on the attained material properties. Polym Int.

[CR29] Zahedi P, Rezaeian I, Ranaei-Siadat SO, Jafari SH, Supaphol P (2010). A review on wound dressings with an emphasis on electrospun nanofibrous polymeric bandages. Polym Adv Technol.

[CR30] Ziabari M, Mottaghitalab V, Haghi AK (2010). A new approach for optimization of electrospun nanofiber formation process. Korean J Chem Eng.

